# A rare case of rectal prolapse after Deloyers procedure in a patient with Hirschsprung’s disease: A case report

**DOI:** 10.1016/j.ijscr.2019.02.021

**Published:** 2019-02-23

**Authors:** Brandon Cheuk-Fung Law, Oswens Siu-Hung Lo

**Affiliations:** Department of Surgery, LKS Faculty of Medicine, The University of Hong Kong, Hong Kong Special Administrative Region, China

**Keywords:** Deloyers, Hirschsprung’s, Adult, Rectal prolapse, Case report

## Abstract

**Introduction:**

Deloyers procedure has been reported in the literature as a viable alternative to the more commonly performed Swenson, Soave and Duhamel methods. As of yet, the long term sequelae of this procedure for patients with Hirschsprung’s disease have not been studied in depth.

**Presentation of case:**

We report the first case in the literatures of a 27-year-old man presenting with rectal prolapse due to colorectal anastomotic intussusception after Deloyers procedure for Hirschsprung’s disease.

**Discussion:**

Few studies with low case volume have been performed investigating the long term sequelae of Deloyers procedure as a mainstay in patients undergoing operative treatment for Hirschsprung’s disease. This procedure allows for preservation of a longer segment of colon, in turn potentially improving absorption and continence compared to other methods. Studies are limited and as of yet the viability of Deloyers as a mainstay of treatment for Hirschsprung’s disease is inconclusive.

**Conclusion:**

We report the first adult case of prolapsed colorectal anastomotic intussusception after Deloyers procedure for Hirschsprung’s disease. Further study is required to delineate long-term complications and viability of this method in these patients.

## Highlights

• Deloyers procedure reported as a colonic salvage procedure after extensive left colon resection.
• Deloyers procedure has been used in long segment Hirschsprung’s disease.
• First case of rectal prolapse after Deloyers procedure for Hirschsprung’s disease.
• Potential complication after Deloyers procedure for colorectal cancer in adult patients.
• Further study required to ascertain long term sequelae in this group of patients.

## 1 Introduction

Hirschsprung’s disease is a congenital disease characterized by aganglioniosis of the colon [1]. Patients commonly present in the first 2 months of life, with symptoms of constipation, vomiting, diarrhea and abdominal pain. Surgery is often indicated, particularly in patients with enterocolitis, bowel obstruction or megacolon [2]. The disease is subdivided into short-segment and long-segment, depending on the extent of bowel involved. In some cases, obstruction progresses to intestinal perforation, leading to mortality. Commonly performed modes of operative treatment for Hirschsprung’s disease include the Swenson, Duhamel and Soave methods [[3], [4], [5]]. The goal of these procedures is to achieve anastomosis between functioning bowel to anus. Choice of procedure and length of resection depends varies depending on patient, disease and surgeon factors.

Deloyers procedure was first presented by the Belgian surgeon Lucien Deloyers in 1963 [6]. After resection of the diseased segment of sigmoid and/or left colon is performed, the transverse or right colon is fully mobilized and anastomosed to the rectum or the anus in an isoperistaltic manner. Transection of the right colic or middle colic arteries is then performed, depending on the remaining length of viable bowel. The right colon is then flipped 180° about its original axis, with the ileocolic pedicle being the axis of rotation. The caecum is sutured to the hepatic flexure. The ascending colon occupies the right paracolic gutter (in the opposite direction) and is sutured to the parietal peritoneum. This procedure allows for preservation of healthy colon after extensive left colonic resection, as opposed to an ileorectal or ileoanal anastomosis [7].

In Deloyers’ initial series, this procedure was performed on eleven patients aged 17–44. These patients suffered from ulcerative colitis, megacolon, dolichocolon, and colonic polyposis [6]. Deloyers procedure has since been reported in the literature as a viable alternative in patients with Hirschsprung’s disease [8,9]. It is nowadays used as a salvage procedure after extensive resection for colorectal cancer [7].

We report a case of an adult patient with history of Hirschsprung’s disease and presenting with rectal prolapse. The patient was initially operated on at 2 years of age, before presenting at our hospital 26 years later. Eventually, laparotomy revealed evidence of a Deloyer’s procedure having been performed.

This presented work has been reported in line with the SCARE criteria [10].

## 2 Case

A 27-year-old Chinese gentleman presented to our colorectal surgery clinic with a one year history of progressively worsening rectal prolapse. He reported a history of Hirschsprung’s disease with an unknown operation performed at 2 years of age. He had a laparotomy and adhesiolysis for intestinal obstruction at age 13. No other significant past medical or mental illness was reported.

**Fig. 1:**
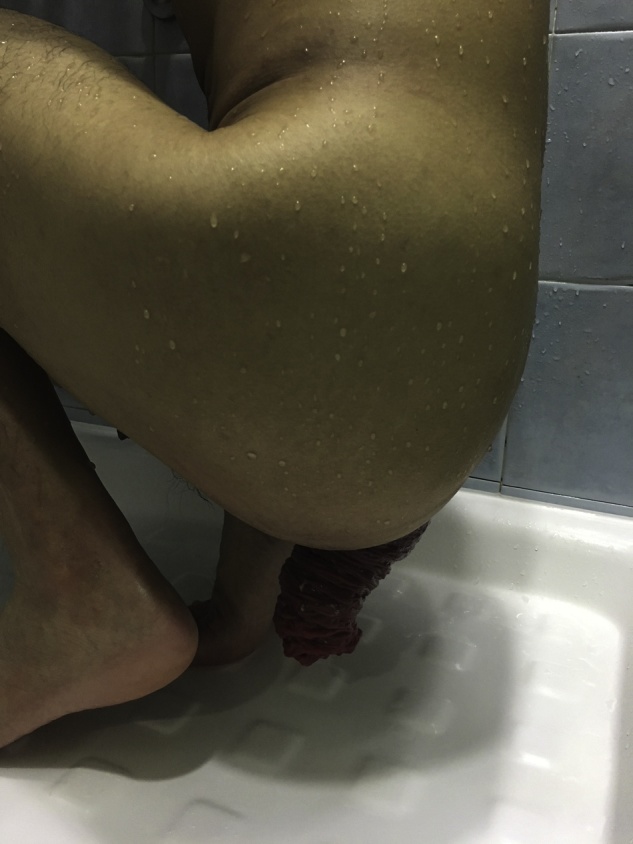
Full thickness rectal prolapse prior to surgery.

The patient complained of a full thickness, completely reducible rectal prolapse occurring after defecation (Fig. 1). He had daily bowel opening and no fecal incontinence. There was no associated abdominal pain, proctalgia or rectal bleeding. Physical examination of the abdomen revealed right transverse and midline abdominal scars. Anal tone was normal on digital rectal examination. There was no descent of the perineum on straining.

**Fig. 2:**
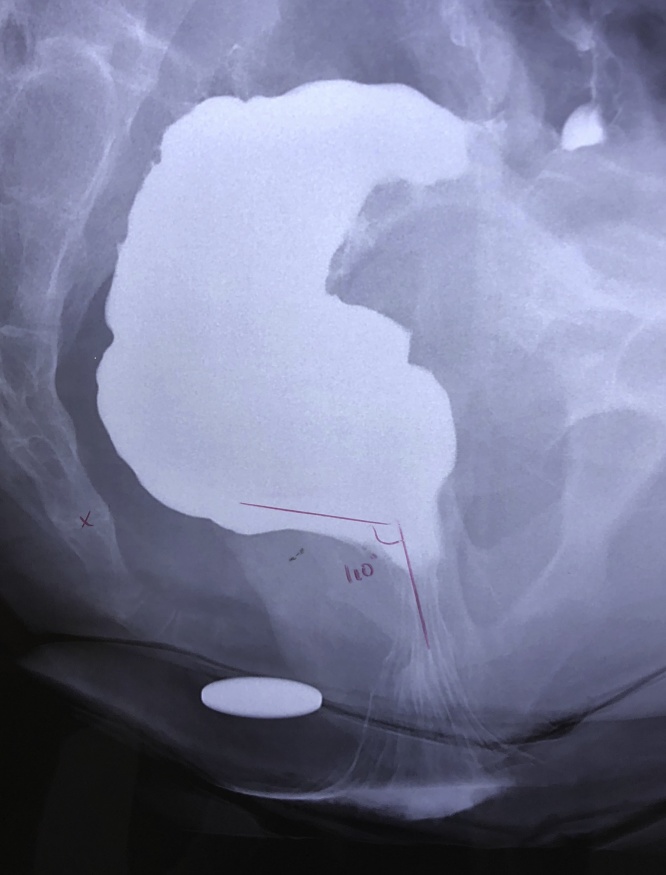
Preoperative contrast defaecography.

Initial workup consisted of a colonoscopy and contrast defaecography. At colonoscopy, a blind end was encountered at 25 cm. A suspected end-to-side ileocolic anastomosis was seen at 2–3 cm distal to the blind end. The scope failed to pass through this suspected anastomosis. Defaecography showed a 5 cm antero-posterior diameter rectal prolapse. It measured 2.5 cm in the cephalo-caudal dimension. There was no intra-rectal intussusception or anterior rectocele. The anorectal angle was 2 cm below the pubococcygeal line (Fig. 2).

**Fig. 3:**
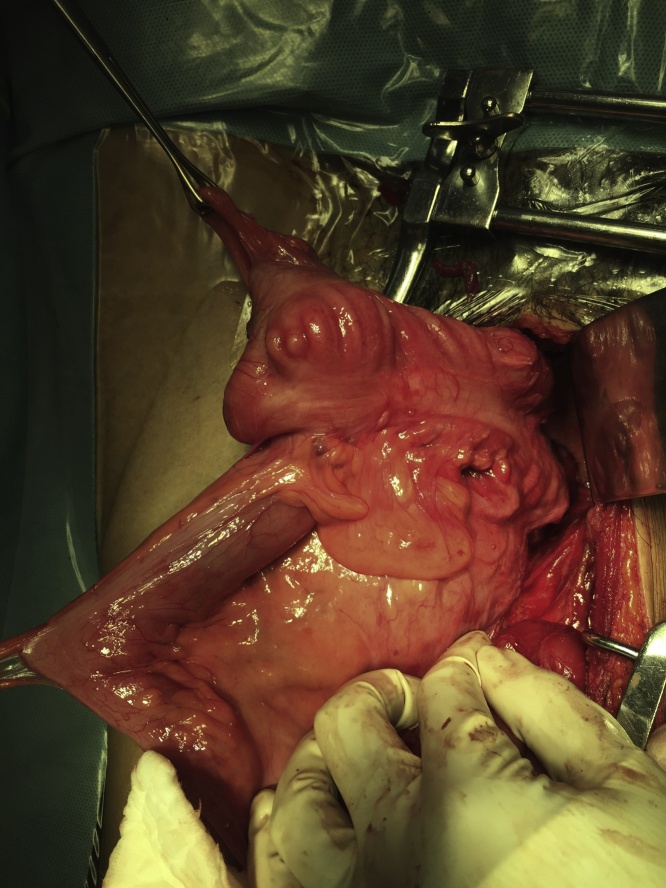
Intraoperative findings of rotated ascending colon with colorectal anastomosis.

**Fig. 4:**
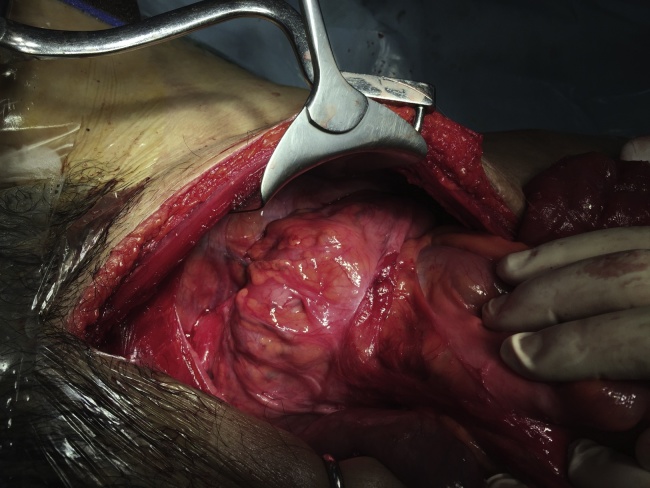
Caecum anchored to parietal peritoneum at right upper quadrant after caecopexy.

Abdominal rectopexy was offered after workup but the patient opted for observation at the time as he worried about the possible surgical complications, like sexual dysfunction. After 11-year regular follow up, he finally agreed for operation due to difficulty in reducing the prolapse completely. Initially, laparoscopic rectopexy was attempted but failed due to dense intraabdominal adhesions. After conversion into laparotomy and adhesiolysis, an isoperistaltic ascending colorectal anastomosis was found at the peritoneal reflection. The right colon was rotated and freely mobile with a long mesentery and minimal retroperitoneal attachment (Fig. 3). The patient’s rectal prolapse was diagnosed to be an anal protrusion of this colorectal anastomotic intussusception, compatible with having had a Deloyers procedure in his youth. Therefore, the caecum was fixed to the parietal peritoneum of right upper quadrant with nonabsorbable polypropylene sutures (Fig. 4). The patient was then discharged after three weeks of postoperative ileus. No recurrence of prolapse was reported on more than two years of follow up.

## 3 Discussion

Deloyers procedure was first described in 1964 [6]. It involves an anastomosis between the right or transverse colon and the rectum or anus after a complete mobilization and rotation of the right colon. The ileocaecal valve and ileocolic artery are preserved. Advantages for this procedure include the preservation of large bowel function and continence after extensive colectomy for long segment Hirschsprung’s disease or colorectal cancer [7]. However, there is a risk of venous ischaemia due to torsion of the vascular pedicle in this group of patients [11]. In contrast to other commonly used methods operative methods, only small studies have been identified regarding the long term sequelae of Deloyers procedure for Hirschsprung’s disease [8,9].

As this is the first case of prolapse presenting after a Deloyer’s procedure for Hirschsprung’s disease, we are unable to conclude that this is a common long term complication of the procedure. As complications exist for each method of operative treatment, further study could be warranted to identify the true advantages and risks of this method compared to more traditional management. Moreover, the operative approach has been used for extensive left colectomy in patients with colorectal cancer. Therefore, all colorectal surgeons should be familiar with the management if this rare complication occurs in this group of patients.

## 4 Conclusion

This is the first reported case of rectal prolapse after Deloyers procedure in the literatures. Although there are limited studies investigating the long-term sequelae of this method, all colorectal surgeons should be familiar with the management once this rare complication presents in these patients.

## Conflict of interest

None.

## Sources of funding

None.

## Ethical approval

This case report is exempt from institutional review board at our institution.

## Consent

Written informed consent was obtained from the patient for publication of this case report and accompanying images(pre and perioperative photographs). A copy of the written consent is available for review by the Editor-in-Chief of this journal on request.

## Author contribution

BCF Law – acquired and interpreted the data and drafted the manuscript.

OSH Lo – performed the operation, followed up the patient and revised the manuscript.

Both authors read and approved the final manuscript

## Registration of research studies

N/A.

## Guarantor

Oswens Siu-Hung LO

MBBS (HK), MRCS (Ed), FRCSEd (Gen), FHKAM (Surgery)

Division chief of colorectal surgery

Department of Surgery, LKS Faculty of Medicine, The University of Hong Kong, Hong Kong SAR, China.

## Provenance and peer review

Not commissioned, externally peer-reviewed.
